# Crystalline covalent organic polymer as an effective zincophilic protective layer to boost the performance of aqueous zinc-ion batteries

**DOI:** 10.1039/d5sc08630b

**Published:** 2026-03-06

**Authors:** Xin Wang, Yuchan Zhang, Lei Zhang, Qianfeng Gu, Qi Liu, Yang Ren, Chun-Sing Lee, Qichun Zhang

**Affiliations:** a Department of Materials Science and Engineering, City University of Hong Kong Hong Kong SAR 999077 P. R. China qiczhang@cityu.edu.hk; b Department of Physics, City University of Hong Kong Hong Kong SAR 999077 P. R. China; c Department of Chemistry, Center of Super-Diamond and Advanced Films (COSDAF) & Hong Kong Institute of Clean Energy, City University of Hong Kong Hong Kong SAR 999077 P. R. China; d City University of Hong Kong Shenzhen Research Institute Shenzhen Guangdong Province 518057 P. R. China

## Abstract

Developing an effective layer to isolate zinc anodes from electrolytes, stop side reactions and help uniform zinc deposition in aqueous zinc-ion batteries (AZIBs) is very important and highly desirable. However, most reported protective layers have less effective ion transport channels and poor zincophilicity, leading to short cycling lifetimes. To address this issue, we try to employ tetrathiafulvalene (TTF) derivatives as building units to construct single crystals of new polymers for such protection because these polymers can provide ordered ion-transport channels and strong interactions between sulfur and zinc species. Here, single crystals of a one-dimensional TTF-based organic polymer with square-wave-shaped chains, designated as CityU-51, have been prepared through the assembly of 4,4′,5,5′-tetra(isoquinolin-6-yl)-2,2′-bi(1,3-dithiolylidene) and 1,4-bis(benzodioxaborole) benzene *via* the formation of B–N bonds. CityU-51 exhibits high zincophilicity as the TTF moieties interact strongly with zinc species, demonstrating exceptional performance in guiding uniform zinc deposition. Remarkably, the as-fabricated AZIBs utilizing CityU-51 as an anode protective layer exhibit an ultra-long lifespan exceeding 6300 hours at 1 mA cm^−2^. Moreover, the batteries can continuously work over 6000 hours even at an ultrahigh current density of 30 mA cm^−2^. A high capacity of 268.63 mAh g^−1^ of a full cell is observed, and a stable capacity retention ratio of 97% is maintained over 500 cycles. This study underscores the significance of functional B–N polymers and offers a novel coating option for dendrite-free anodes in AZIBs.

## Abstract 2

Single crystals of a 1D tetrathiafulvalene-based covalent organic polymer (CityU-51) serve as anode protective layer in aqueous Zn-ion batteries, achieving an ultra-long lifespan exceeding 6300 h at 1 mA cm^2^ and over 6000 h at 30 mA cm^2^.

## Graphical abstract

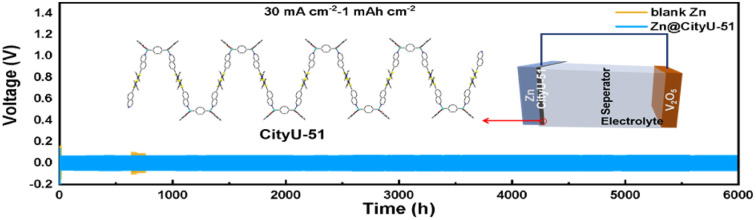

## Introduction

Rechargeable aqueous Zn-ion batteries (AZIBs) present a promising solution for energy storage due to their abundant resources, environmental friendliness, and high safety.^1–4^ However, the practical development of AZIBs is strongly impeded by challenges associated with Zn anodes, such as dendrite growth and side reactions, which degrade overall performance.^5,6^ Various strategies have been proposed to address these issues, with surface protection emerging as a facile yet effective approach to boost the electrochemical stability.^7^ Protective layers constructed on zinc anodes effectively isolate them from the electrolyte, preventing side reactions and facilitating uniform zinc deposition.^8,9^ To date, diverse agents such as metal oxides, carbon, composites, and polymers^10^ have been employed for surface modifications. Among them, covalent organic polymers (COPs) are particularly desirable for Zn anode protection due to their abundance of functional groups, which can regulate Zn^2+^ migration through coordination or adsorption with ions, thereby contributing to the efficient layer zincophilicity.^11–14^

However, many conventional COPs demonstrate low Zn^2+^ diffusion and inhomogeneous zinc deposition due to the lack of strong zincophilic units and effective ion transport channels.^15^ Moreover, because conventional polymers are amorphous or polycrystalline powders due to poor solubility and chain entanglements, it is challenging to understand their precise structures as well as the chain arrangements in space.^16^ Therefore, achieving single-crystal COPs is highly desirable, as they provide insights into molecular arrangements and precise intermolecular interactions,^17,18^ thereby aiding our understanding of how functional groups and stacking modes influence protective performance in AZIBs.

As an important family member of crystalline COPs, dative boron–nitrogen (B–N) polymers have emerged as a significant research focus due to the reversible nature of B–N linkages, facilitating the facile generation of polymer single crystals.^19,20^ These polymers are distinguished by their unique bonding characteristics: the B–N linkages exhibit moderate bonding strength (∼100 kJ mol^−1^) with high reversibility,^21^ combining the dynamic nature of hydrogen bonds (10–40 kJ mol^−1^)^22^ with the robustness of common covalent linkages like azodioxy and amine (100–400 kJ mol^−1^).^23,24^ This balance allows for error self-correction during crystallization through reversible bond breaking and reforming, thereby enhancing their structural integrity and realizing single crystals.^25–27^ Benefitting from solution processability and diverse organic ligands, B–N polymers have become ideal candidates for solvent separation and photocatalysis applications.^28–30^ Our previous studies have shown their promising performance in photodetection and energy storage,^31–33^ motivating us to further develop multifunctional B–N polymers and explore their potential applications.

Introducing active groups that interact with Zn ions into B–N polymers is of interest, as it can significantly enhance performance in AZIBs. The flexible B–N bonding facilitates the formation of polymer single crystals, aiding in understanding their roles within battery systems. Tetrathiafulvalene (TTF) is a well-known electron donor that is usually employed to prepare organic metals and superconductors.^34,35^ Its sulfur-rich segments efficiently react with metal ions, making it promising for electrode materials that can boost battery electrochemical performance.^36^ Regions around S atoms in TTF units are attractive to anions, which might guide the uniform deposition of zinc ions.^37^ This has inspired us to explore its application potential in AZIBs further. Aligned, one-dimensional (1D) chains could assemble into a dense, highly ordered lattice. This structure facilitates a homogeneous distribution of Zn^2+^-selective ion pathways *via* the action of TTF functional groups, which promote Zn^2+^ desolvation and ensure its uniform deposition. Therefore, in this research, we developed a new 1D TTF-containing B–N polymer single crystal, CityU-51, utilizing the pyridine ligand 4,4′,5,5′-tetra(isoquinolin-6-yl)-2,2′-bi(1,3-dithiolylidene) (namely TTF-iqn) and the boron donor 1,4-bis(benzodioxaborole) benzene (BACT). The active sites of CityU-51 enable efficient interactions with zinc ions, demonstrating promising potential in the protection of Zn metal anode. The CityU-51-modified anode exhibited significantly improved stability and cycling life, operating effectively for over 6300 hours at 1 mA cm^−2^, and could still work well even at an ultrahigh current density of 30 mA cm^−2^ for over 6000 hours. A high capacity of 268.63 mAh g^−1^ of a full cell was observed, and a stable capacity retention ratio of 97% was maintained over 500 cycles. This advancement highlights the effectiveness of integrating TTF into B–N polymers to enhance AZIB performance, paving the way for realizing more stable and efficient energy storage.

## Results and discussion

**Fig. 1:**
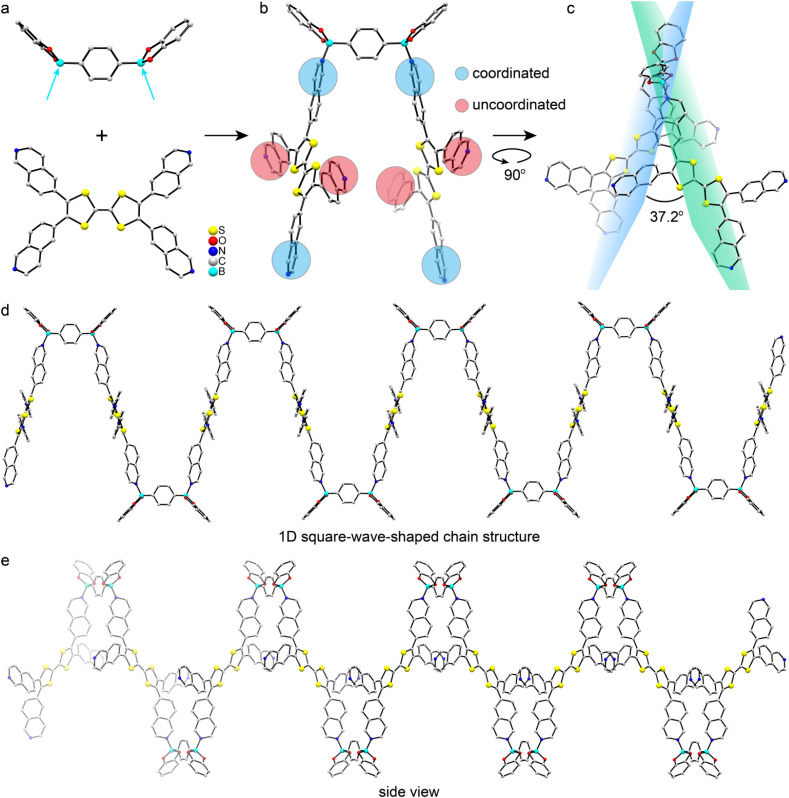
(a) and (b) Schematic illustration of preparation process of CityU-51. One BACT unit and two TTF-iqn moieties to which it is connected in CityU-51. Thermal ellipsoids are drawn at the 50% probability level. Toluene molecules are omitted for clarity. (c) The dihedral angle of two neighboring TTF-iqn molecules. (d) A square-wave-shaped chain in CityU-51. Hydrogen atoms are omitted for clarity. (e) Side view of the square-wave shaped chains.

**Fig. 2:**
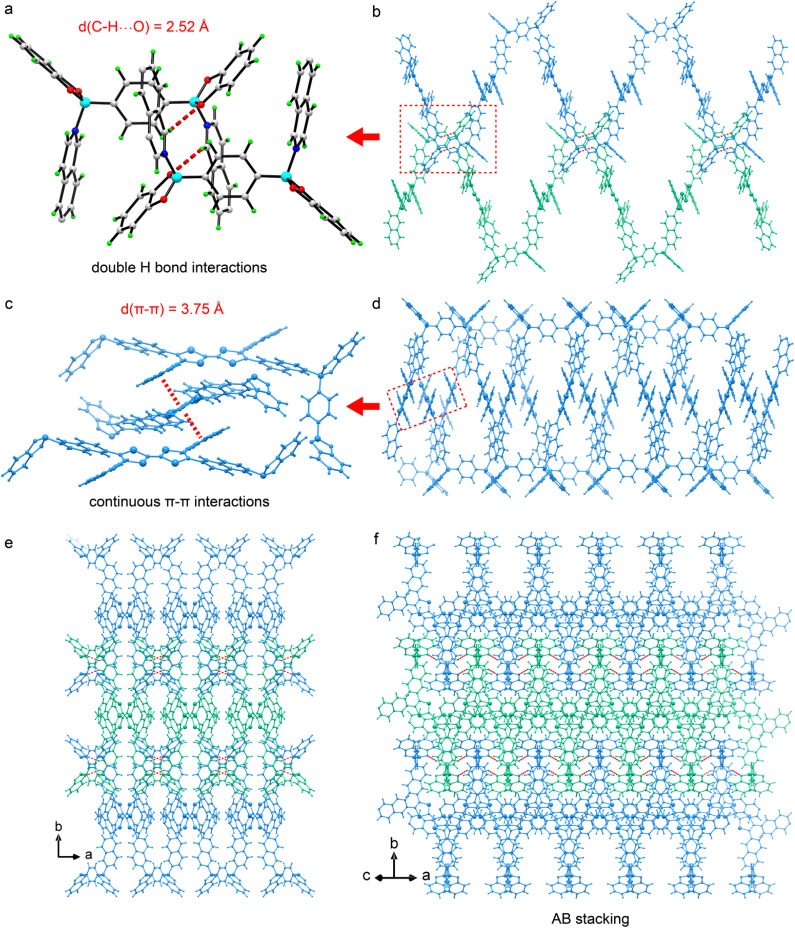
(a) and (b) Illustration of the hydrogen bonding interactions (represented in red dashes) between neighboring chains. (c) and (d) Illustration of continuous π–π interactions in CityU-51. (e) Illustration of a framework in CityU-51 constructed by C–H⋯O hydrogen bonds interactions, represented in red dashes. (f) Illustration of the stacking pattern in CityU-51.

The monomers TTF-iqn and BACT were synthesized following the reported methods with slight modifications (Fig. S1 and S2).^38–40^ Through B–N-bonding-driven self-assembly between TTF-iqn and BACT, single crystals of green one-dimensional (1D) polymer CityU-51 (CCDC 2450236), with a size of several hundred micrometers, were obtained *via* high-temperature evaporation of a toluene-methanol mixed solution (Fig. S3). The strong steric hindrance of isoquinoline allows only two diagonal pyridine moieties of TTF-iqn to react with two boron atoms from BACT, resulting in a 1D polymer chain (Fig. 1a and b). Fig. S4 illustrates one asymmetric unit of CityU-51, comprising half a TTF-iqn, half a BACT, and half a toluene molecule (Table S1), where TTF-iqn and BACT are linked by B–N bonds with a length of 1.637 Å (Table S2), consistent with previous studies (1.627 to 1.691 Å).^41^ One BACT connects two neighboring TTF-iqns, with its benzene rings adopting a *cis* arrangement, and the dihedral angle between the two TTF-iqn molecules is 37.2° (Fig. 1c). The main bond angles are also summarized in Table S3. Additionally, both TTF-iqns are positioned on the same side of BACT, forming a square-wave-shaped chain instead of the typical zig-zag configuration (Fig. 1d and e). The width of one chain is 29.07 Å. Consequently, hydrogen interactions (about 2.52 Å) between oxygen atoms (BACT) and hydrogen atoms (isoquinoline moieties from TTF-iqn) among neighboring chains stabilize the overall packing of CityU-51 (Fig. 2a and b). Continuous π–π interactions (∼3.75 Å) between uncoordinated isoquinoline moieties from two adjacent chains are also observed and contribute to structure stabilization (Fig. 2c and d). Additionally, intermolecular hydrogen bonding generates a 3D supramolecular framework, which adopts an AB stacking pattern (Fig. 2e and f). These strong interactions among polymer chains render CityU-51 insoluble in toluene and water even at high temperatures, unlike some zig-zag B–N polymers that can be redissolved.^42,43^

Powder X-ray diffraction (PXRD) analysis was conducted to assess the phase purity of the synthesized products. Fig. S5 presents the measured PXRD pattern of the products alongside a simulated pattern based on the single-crystal structure of CityU-51, which shows good agreement, indicating the absence of impurities or side products during sample preparation. Small blue shift at low angles was attributed to lattice contraction originating from disorder within the crystal structure, where the atomic vibration was strong.^44^ Thermogravimetric analysis (TGA) reveals that the initial weight loss is approximately 8.2%, consistent with the weight ratio of toluene molecules in CityU-51 (Fig. S6). Considering solvent loss, CityU-51 can be regarded as stable up to 265 °C. UV-vis absorption spectra of CityU-51, BACT, and TTF-iqn were also analyzed (Fig. S7). BACT exhibits minimal absorption below 450 nm, while TTF-iqn primarily absorbs in the visible light region. In contrast, CityU-51 displays two broad peaks at 900 and 1200 nm, likely due to its nested interlocking structure. The formation of B–N linkages is further confirmed by Fourier Transform Infrared (FTIR) spectroscopy (Fig. S8). Peaks at 1068 cm^−1^ and 1030 cm^−1^ correspond to B–N bonds, while peaks at 780 cm^−1^ and 1624 cm^−1^ are attributed to C–S and C=C bonds from TTF-iqn, respectively.

**Fig. 3:**
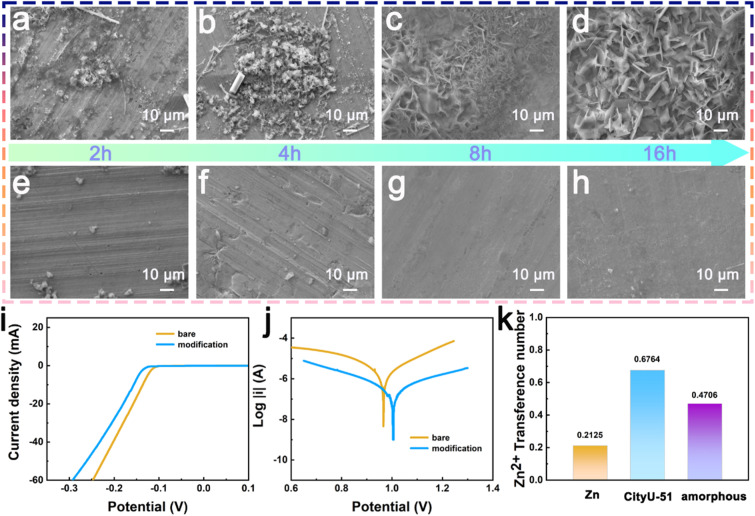
The SEM images at different deposited time of (a–d) blank Zn metal and (e–h) modified Zn metal; (i) The LSV curves, (j) the Tafel curve, and (k) the Zn^2+^ transference number of unmodified zinc electrodes and the electrodes modified with either single-crystalline CityU-51 or amorphous polymer layers.

As sulfur-rich TTF can interact with metal ions efficiently, CityU-51 is considered an ideal candidate for protecting zinc electrodes in AZIBs. Specifically, the C–S bonds of TTF within CityU-51 exhibit intense interactions with zinc ions. The mortise-tenon structures of CityU-51 present a periodic arrangement of TTF units, which promotes the uniform deposition of zinc ions in AZIBs. Additionally, the excellent stability of CityU-51 in aqueous environments has prompted an investigation into its performance for modifying zinc metal anodes. Fig. S9 and S10 illustrate the efficient and uniform deposition of the CityU-51 layer on the Zn anode, as evidenced by the FTIR spectrum of Zn@CityU-51 confirming the presence of characteristic functional groups (B–N and C–S bonds), while the EDS mapping visually demonstrates its homogeneous surface coverage across the electrode surface. Fig. S11 suggested the layer thickness of 3 µm through a cross-sectional scanning electron microscopy (SEM) image. Experimental results reveal significant differences in zinc ion deposition on bare *versus* modified zinc surfaces, as illustrated in the SEM images (Fig. 3). Fig. 3a depicts the morphology of zinc plating on bare zinc metal after 2 hours in a 2 M Zn(CF_3_SO_3_)_2_ electrolyte at a current density of 1 mA cm^−2^. Initially, few sharp flakes were observed on the flat surface. As the deposition time increased, both the quantity and size of these flakes changed markedly. Ultimately, zinc dendrites formed, expanding to cover nearly the entire surface of the zinc metal. After longer deposition times, when the deposition amount reached 16 mAh cm^−2^, the dendrite size increased to 15–25 µm, and their shapes became sharper (Fig. 3d). In contrast, SEM images of CityU-51-modified zinc electrode (Zn@CityU-51), presented in Fig. 3e–h, show that a smooth surface remained throughout the deposition process. The modified zinc metal retained its flatness without dendrite formation, even after extended deposition times. Their cross-sectional SEM images also supported the dendrite inhibition on the CityU-51-modified Zn metal (Fig. S12). The presence of dendrites on unmodified zinc indicates side reactions at the interface, which can lead to hydrogen evolution and reduced battery lifespan. Furthermore, large dendrites pose a risk of penetrating the separator, potentially causing short circuits. Compared to unmodified zinc, the modified zinc anode with a flat surface demonstrates that the CityU-51 layer effectively inhibits dendrite formation and minimizes interfacial side reactions. To evaluate the impact of CityU-51, linear sweep voltammetry (LSV) measurements were conducted (Fig. 3i). The Zn@CityU-51 anode exhibited smaller current responses, indicating that the CityU-51 layer mitigates hydrogen release.

Additionally, Tafel curves for both unmodified zinc and Zn@CityU-51 electrodes in a 2 M Zn(CF_3_SO_3_)_2_ electrolyte were analyzed to assess corrosion suppression (Fig. 3j). The Zn@CityU-51 electrode demonstrated a higher self-corrosion potential and a lower self-corrosion current compared to bare zinc, suggesting that the CityU-51 layer effectively reduces corrosion rates and enhances corrosion resistance. Electrochemical impedance spectroscopy (EIS) measurements were performed to further investigate the resistance of the Zn@CityU-51 electrode before and after cycling (Fig. S13). A fitted equivalent circuit model was employed to analyze the electrochemical data employing an auto setup. The fitting impedance increased slightly from 1200 Ω to 1250 Ω after cycling, indicating a change in the electrochemical behavior of the modified electrode. This increase is attributed to the initial SEI formation of between electrolytes and zinc electrodes. From the current–time curves and EIS results (Fig. S14), the corresponding transference numbers were determined for zinc electrodes modified with either single-crystalline CityU-51 or an amorphous polymer layer. The electrode modified with CityU-51 exhibited a transference number of 0.6764, significantly higher than the value of 0.4706 for the amorphous polymer-modified electrode and 0.2125 for the unmodified zinc electrode (Fig. 3k). This quantitative hierarchy strongly indicates that the ordered single-crystalline CityU-51 exhibits high kinetics of Zn^2+^, thereby facilitating more efficient and selective Zn^2+^ migration. This conclusion is further supported by the electrochemical performance of symmetric cells, where the amorphous polymer layer resulted in markedly inferior cycling stability and a shorter lifespan (350 h at 1 mA cm^−2^ and 1 mAh cm^−2^, Fig. S15), underscoring the superior protective efficacy of the ordered crystalline structure. Additionally, chronoamperometry (CA) was performed to study zinc nucleation behavior during electrodeposition (Fig. S16). At a constant overpotential of −150 mV, the current for both bare Zn and Zn@amorphous symmetric cells changed substantially and continued to decay over 300 s. In contrast, the current for the Zn@CityU-51 symmetric cell stabilized within only 5 s. This suggests that the prolonged, two-dimensional diffusion of Zn^2+^ ions accompanied by disordered nucleation on bare Zn has been effectively regulated into a shorter, three-dimensional diffusion process with uniform nucleation under the CityU-51 coating. Furthermore, the CityU-51 layer prevents electrolyte penetration into the zinc anode surface, thereby enhancing the transport kinetics of Zn ions.

**Fig. 4:**
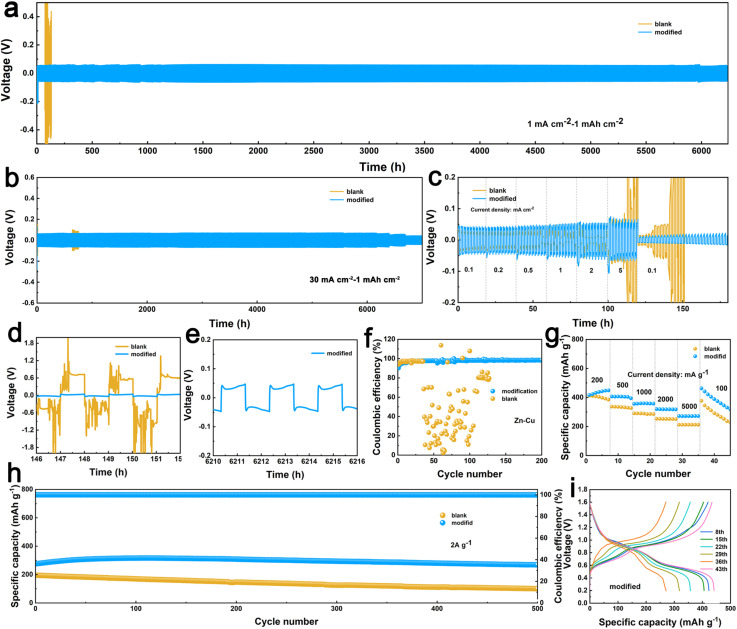
The cycling performance of Zn@CityU-51-Zn batteries at (a) 1 mA cm^−2^, (b) 30 mA cm^−2^; (c) the rate capability and (d and e) the amplified cycling curves of Zn@CityU-51-Zn batteries at 1 mA cm^−2^; (f) the coulombic efficiency of Zn@CityU-51-Cu batteries; (g) the rate capability, (h) the cycling performance at 2 A g^−1^ and (i) the charging/discharging profiles of V_2_O_5_//Zn@CityU-51-Cu batteries.

To comprehensively investigate the electrochemical reversibility of the protective CityU-51 layer on zinc electrodes, experiments were conducted using both Zn–Zn symmetric batteries and Zn–Cu asymmetric batteries at various current densities (Fig. 4). The Zn@CityU-51-Zn batteries operating at 1 mA cm^−2^ demonstrated that the Zn@CityU-51 electrode exhibited superior cycling stability, lasting over 6300 hours, in contrast to the bare zinc electrode, which failed after only 100 hours (Fig. 4a). Furthermore, the Zn@CityU-51-Zn batteries showed only initial signs of degradation after 7000 hours of continuous cycling (Fig. S17). This significant difference underscores the effectiveness of the CityU-51 layer in improving Zn^2+^ transmission dynamics and suppressing interfacial side reactions. Fig. 4c presents the rate capability of the batteries. As the current density increased from 0.1 mA cm^−2^ to 5 mA cm^−2^, the voltage of the Zn@CityU-51 electrode increased gradually. In contrast, the bare zinc electrode experienced a sharp potential rise to 0.2 V due to interfacial failure, highlighting the enhanced stability of the modified anode. Fig. S18 and 4b present further experiments conducted at higher current densities of 10 and 30 mA cm^−2^, which exhibited excellent stability as well, indicating that the CityU-51 layer not only improved conductivity but also lowered deposition potential, thereby lowering nucleation barriers. The polarization voltage at 1, 10, and 30 mA cm^−2^ showed slight difference, with the measured values of 0.03, 0.05, and 0.08 V, respectively (Fig. 4e and S19). Notably, under extremely harsh conditions (30 mA cm^−2^), the battery maintained stable operation for over 6000 hours, surpassing most previously reported protection strategies for zinc anode (Table S4).^45–59^Fig. 4d and e depict amplified plating and stripping curves, clearly demonstrating the failure of the unmodified battery. The Zn–Cu batteries were employed to assess coulombic efficiency (CE), and the Zn@CityU-51-Cu asymmetric battery exhibited higher and more stable CE after 200 cycles compared to the bare Zn–Cu battery, which rapidly declined over 40 cycles due to Zn^2+^ exhaustion at the interface (Fig. 4f). The plating and stripping curves of the Zn–Cu battery in Fig. S20 further support the more stable performance of the modified anode. Additionally, the distinctly different surfaces of the two electrodes after cycling (Fig. S21) reveal that sharp flakes were effectively suppressed following the introduction of CityU-51, indicating a reduction in dendrite growth. Here, X-ray Photoelectron Spectroscopy (XPS) analysis of the bare Zn and Zn@CityU-51 electrodes after cycling was conducted (Fig. S22). For the bare Zn electrode, peaks located at 169.7 eV and 167 eV were attributed to SO_4_^2−^ and SO_3_^2−^ ions from the electrolyte, respectively. While the spectrum for the Zn@CityU-51 electrode was markedly different, featuring peaks at 164.6 eV (S 2p_1/2_) and 162.5 eV, which is a definitive signature of ZnS. This formation of zinc sulfide provides direct evidence of a potent chemical interaction between the CityU-51 coating and zinc ions. Therefore, it can be concluded that the CityU-51 layer provides optimal sites for ion deposition, lowers energy barriers, and suppresses side reactions, thereby significantly enhancing the electrochemical performance of the zinc electrode. Besides, the long-term stability should be attributed to the highly ordered single-crystal structures and the efficient zincophilicity of TTF units.

To further investigate the electrochemical cell performance, we compared the cycling behavior of V_2_O_5_//bare Zn and V_2_O_5_//Zn@CityU-51 batteries (Fig. 4g–i). After pre-cycling, the CityU-51-modified battery exhibited superior stability with a capacity retention ratio of 97% and a higher specific capacity of 268.63 mAh g^−1^ compared to the bare battery (retention ratio of 52% and capacity of 100.9 mAh g^−1^) over 500 cycles at a current density of 2 A g^−1^ (Fig. 4h). The cyclic voltammetry (CV) curves in Fig. S23 reveal distinct cathodic peaks. The first four cycles of the modified electrode exhibited better curve fitting, indicating the enhanced cycling stability. Besides, the anodic peaks of both batteries showed a clear distinction, with the CityU-51-modified battery displaying higher peaks at 0.7 V and 1.0 V by the third cycle, reflecting its superior electrochemical reactivity. The rate capability was evaluated at various current densities (200, 500, 1000, 2000, and 5000 mA g^−1^), as shown in Fig. 4g. The modified battery consistently achieved better specific capacities, ranging from 272.2 to 416.87 mAh g^−1^, compared to the bare battery, underscoring the significance of CityU-51 in enhancing battery performance. Fig. 4i and S24 depict the charging and discharging curves of two batteries at various current densities, which align with the CV peaks observed for V_2_O_5_. Additionally, EIS analysis reveals lower impedance for the Zn@CityU-51//V_2_O_5_ battery compared to the bare counterpart (Fig. S25), indicating improved interfacial and ionic conductivity facilitated by the CityU-51 layer.

## Conclusions

In summary, we have successfully prepared single crystals of a 1D polymer (CityU-51), containing square-wave-shaped chains formed through B–N bonding interactions between TTF-iqn and BACT. Benefiting from sulfur atoms in the TTF moieties that can efficiently bond with zinc species, CityU-51 has been employed as an effective protective layer of anodes in AZIBs, demonstrating exceptional performance. A full cell with the anode covered by CityU-51 exhibited a high capacity of 268.63 mAh g^−1^ and maintained a stable capacity retention ratio of 97% over 500 cycles. Notably, the stability of Zn@CityU-51 anode in the symmetric battery was significantly enhanced, allowing for reliable operation for over 6300 hours at 1 mA cm^−2^. Moreover, the battery could continuously work well even at an ultrahigh current density of 30 mA cm^−2^ over 6000 hours. Our success demonstrated that single crystals of sulfur-containing polymers could have great potential applications in AZIBs.

## Author contributions

Q. Liu, Y. Ren, C. S. Lee, Q. Zhang designed the research. X. Wang and Y. Zhang conducted the experiments. L. Zhang analyzed the crystallographic data. Q. Gu conducted manuscript revision.

## Conflicts of interest

There are no conflicts to declare.

## Supplementary Material

SC-017-D5SC08630B-s001

SC-017-D5SC08630B-s002

SC-017-D5SC08630B-s003

SC-017-D5SC08630B-s004

SC-017-D5SC08630B-s005

## Data Availability

CCDC 2450236 contains the supplementary crystallographic data for this paper.^60^ The authors confirm that the data supporting the findings of this study are available within the article and/or its supplementary information (SI). Supplementary information is available. See DOI: https://doi.org/10.1039/d5sc08630b.
