# Supplementary material for: Crystalline covalent organic polymer as an effective zincophilic protective layer to boost the performance of aqueous zinc-ion batteries
Source: Chem Sci. 2026 Mar 6;17(17):8692–700. doi: 10.1039/d5sc08630b (PMC12974599; doi:10.1039/d5sc08630b)
Supplement: SC-017-D5SC08630B-s002 [file SC-017-D5SC08630B-s002.pdf]

Supplementary Information (SI) for Chemical Science.
This journal is © The Royal Society of Chemistry 2026

Supporting Information

Crystalline Covalent Organic Polymer as an Effective Zincophilic Protective Layer to Boost the Performance of Aqueous Zinc-Ion Batteries

Xin Wang,[1,†] Yuchan Zhang,[1,†] Lei Zhang,[1] Qianfeng Gu[1], Qi Liu,[2] Yang Ren[2], Chun Sing Lee,[3*] Qichun Zhang[1,3,4*]

[1] Department of Materials Science and Engineering, City University of Hong Kong, Hong Kong SAR, 999077, P. R. China.A. B.

[2] Department of Physics, City University of Hong Kong, Hong Kong SAR, 999077, P. R. China

[3] Department of Chemistry, Center of Super-Diamond and Advanced Films (COSDAF) & Hong Kong Institute of Clean Energy, City University of Hong Kong, Hong Kong SAR, 999077, P. R. China.

[4] City University of Hong Kong Shenzhen Research Institute, Shenzhen, Guangdong Province, 518057, P. R. China.

† These two authors have equal contribution

*Email: qiczhang@cityu.edu.hk

1. Materials and methods

All chemicals and solvents were used directly without purification if not specified. PXRD pattern was collected by Rigaku X-ray Diffractometer SmatlabTM 9kW. Thermal analysis was conducted on PerkinElmer Simultaneous Thermal Analyzer (STA) 6000 from 30 to 800 °C at a heating rate of 10 °C min^{-1} under N_2. UV-Vis spectra (300-1500 nm) were measured on Hitachi UH4150 UV-VIS-NIR Spectrophotometer. FTIR spectra (4000 to 400 cm^{-1}) were obtained on a Perkin Elmer Spectrum II.

SCXRD test of **CityU-51** was conducted on the Rigaku X-ray Single Crystal Diffractometer System (Rigaku SmartLab 9kW-Advance) at room temperature using the monochromatized wavelength (Cu Kα) = 1.54184 Å. The crystal structures were solved and refined by full matrix least-squares methods against F2 using the SHELXL-2013-2 program package and Olex-2 software. All non-hydrogen atoms were refined

with anisotropic displacement parameters and hydrogen positions were fixed at calculated positions and refined.

Synthesis of TTF-iqn

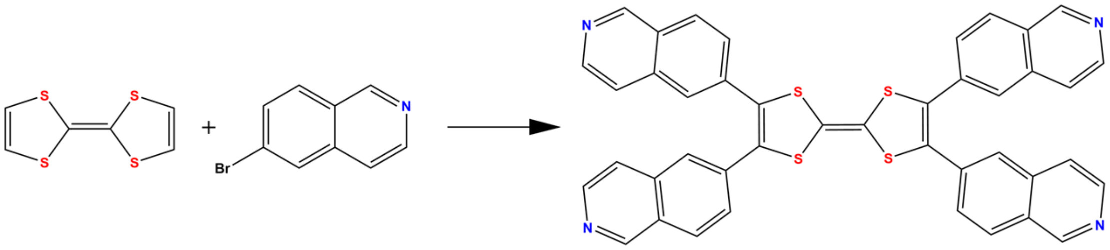

Figure S1. Synthetic route to TTF-iqn.

Palladium acetate (82 mg), tri-tert-butylphosphonium tetrafluoroborate (320 mg), and cesium carbonate (2.40 g) were placed in 100 mL two-neck flask, followed by the addition of 20 mL distilled dioxane under N_2 atmosphere. The suspension was heated at 90 °C for 10 min under argon for activation. Then, 20 mL argon-degassed dioxane including tetrathiafulvalene (300 mg, 1.46 mmol) and 6-Bromoisoquinoline (1.56 g, 7 mmol) were added into the flask. The reaction was stirred under reflux for 72 h. After cooling, the products were extracted from chloroform and purified with silica gel using petroleum ether-dichloromethane as fluid phase.

Synthesis of BACT

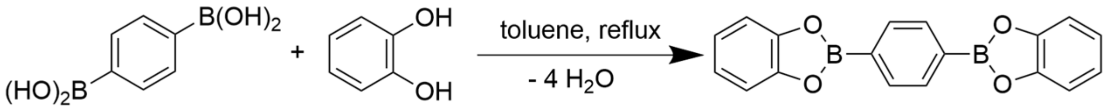

Figure S2. Synthetic route to BACT.

Synthesis of CityU-51

To a mixture of 3 mL toluene and 0.25 mL methanol was added 5 mg of TTF-iqn and 3mg of BACT, followed by ultrasonication to get clear red solution. Evaporation of this solution at 85 °C provided dark-green crystals (**CityU-51**, CCDC 2450236)

Synthesis of amorphous polymer

TTF-iqn (5 mg) and BACT (3 mg) were added to toluene (3 mL). The mixture was heated 85 °C overnight, yielding a red powder.

Synthesis of Zn@CityU-51 anode

9 mg of **CityU-51**, 1 mg of $Zn(CF_3SO_3)_2$, and 1mg of PVDF were added into NMP solvent to obtain a mixture. The mixture was then coated uniformly onto zinc metal (Zn foil: 150 μm) using a 50 μm-coater get a layer of 3 μm, followed by drying at 80 °C in a vacuum oven to obtain the Zn@**CityU-51** anode. Then, the Zn foil was cut into circular pieces with a diameter of 12 mm.

Electrochemical measurements

CR2032 coin cells were assembled to evaluate Zn stripping and plating. Glass microfiber (Whatman, GF/D) served as the separator, while a 2M $Zn(CF_3SO_3)_2$ aqueous solution with 100 μL was used as the electrolyte. The NEWARE battery test system was employed to study the electrochemical performance at room temperature. V_2O_5 was used as the cathode and either Zn or Zn@**CityU-51** as the anode to assemble the full batteries for further testing. The V_2O_5 cathode was prepared by mixing V_2O_5 active material, PVDF binder, and Super P in a weight ratio of 6:2:2. The slurry was stirred for approximately 24 hours to ensure homogeneity and then coated onto the Ti current collector. A vacuum oven was used to dry the slurry at 80 °C for over 12 hours. The LSV, Tafel, EIS and CA tests were executed by electrochemical workstation. All electrochemical measurements were carried out at room temperature.

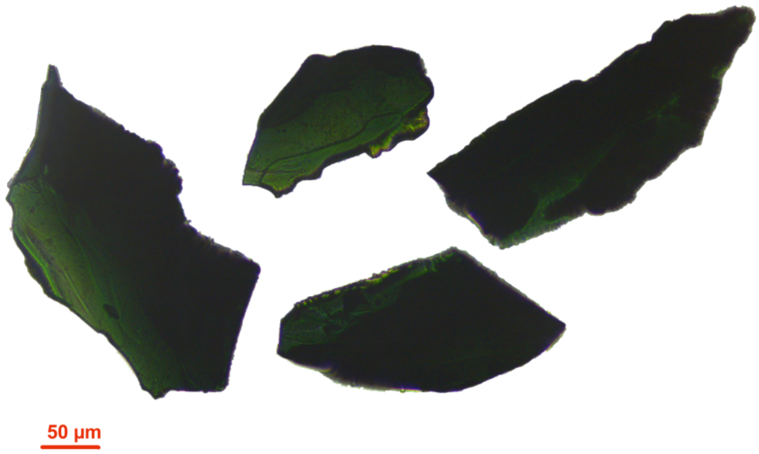

Figure S3. Optical image of **CityU-51** crystals.

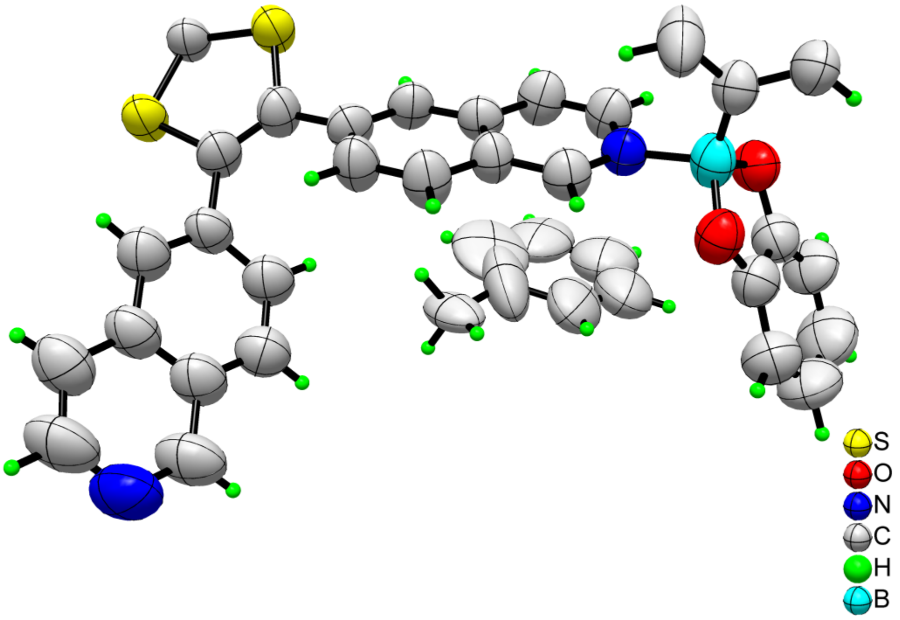

Figure S4. One asymmetric unit of **CityU-51**.

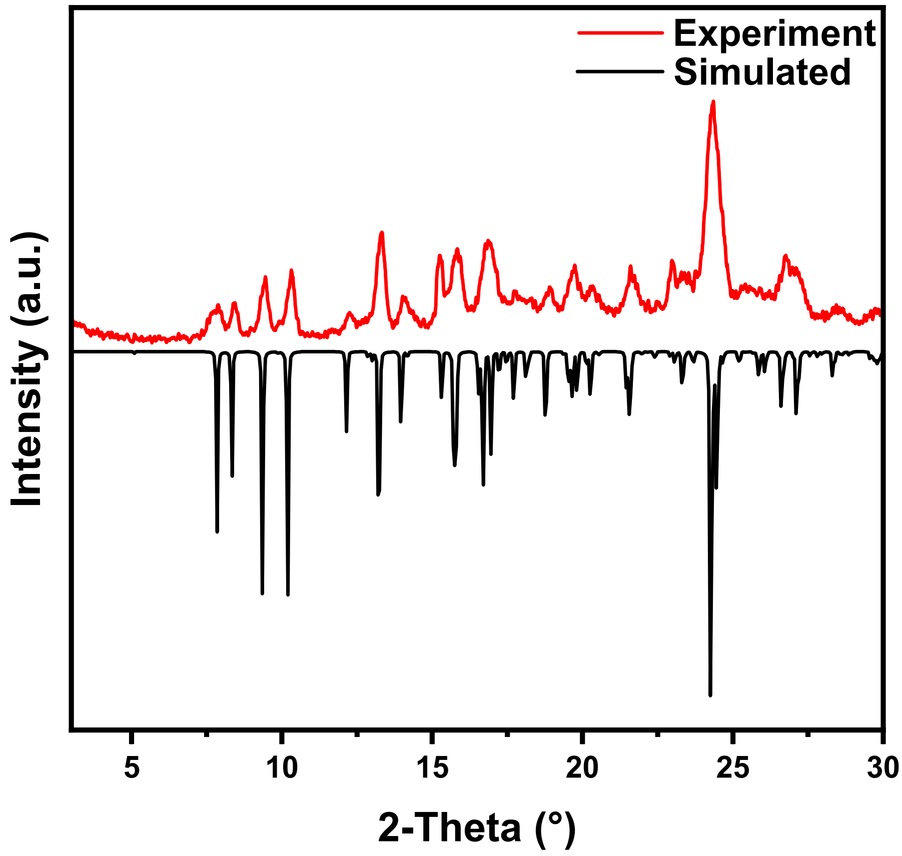

Figure S5. Experimental and simulated PXRD patterns of **CityU-51**.

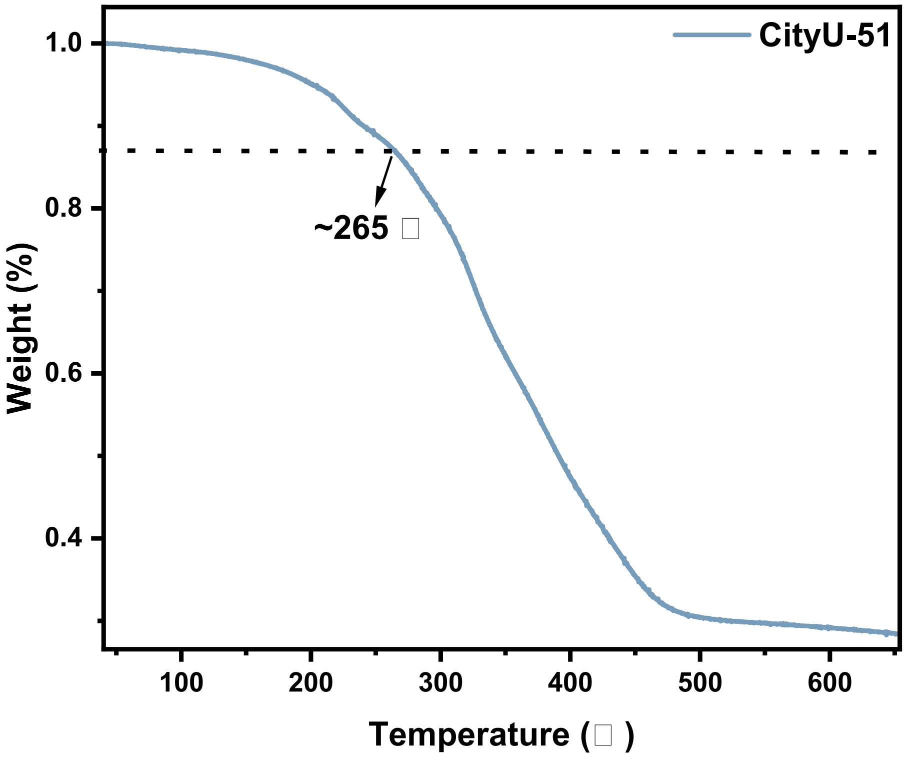

Figure S6. TGA curve of **CityU-51**.

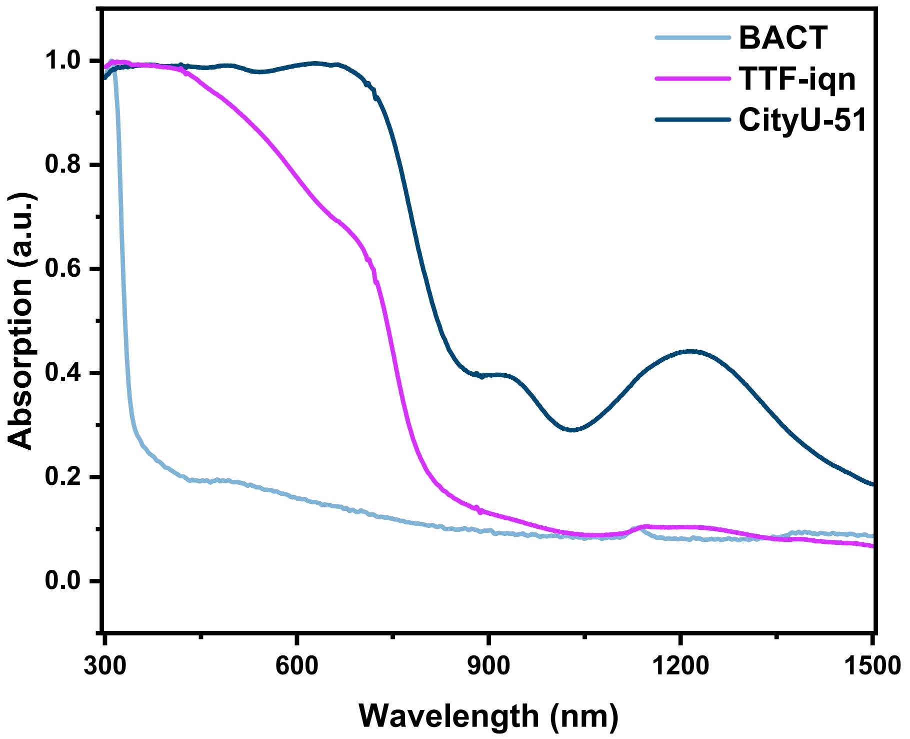

Figure S7. UV-Vis spectra of **CityU-51**, TTF-iqn and BACT.

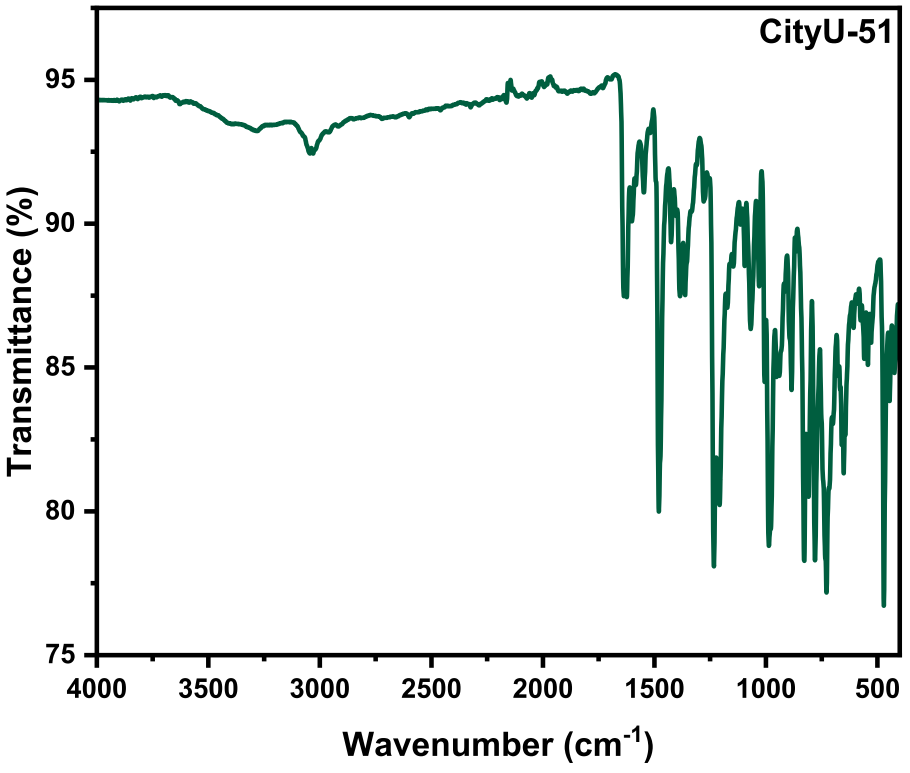

Figure S8. FTIR spectrum of **CityU-51**.

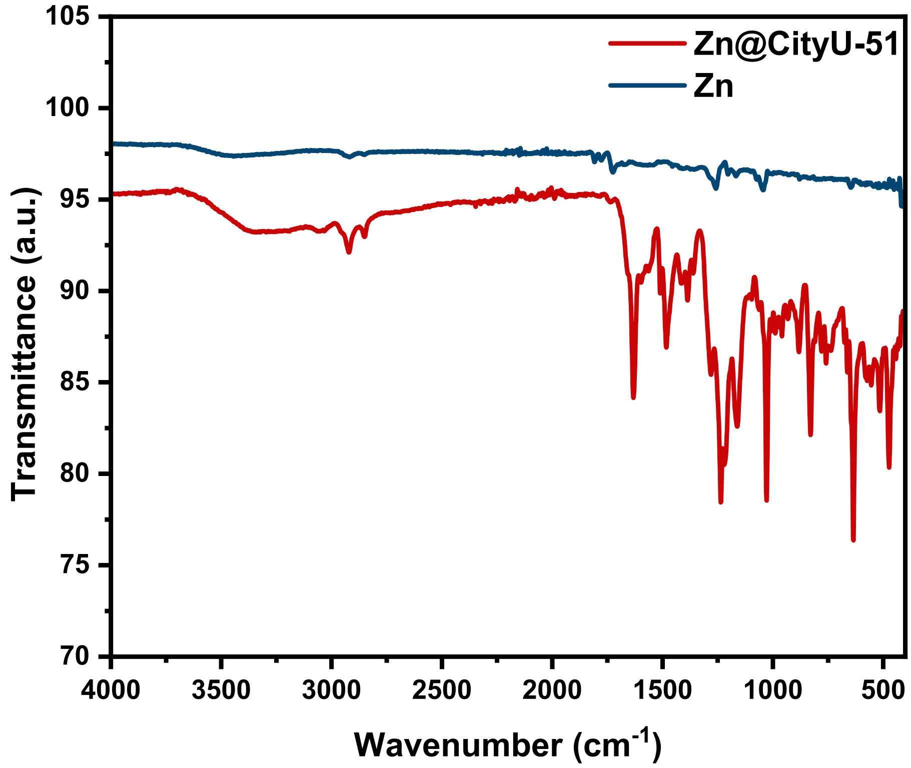

Figure S9. FTIR spectra of Zn metal and Zn@**CityU-51**.

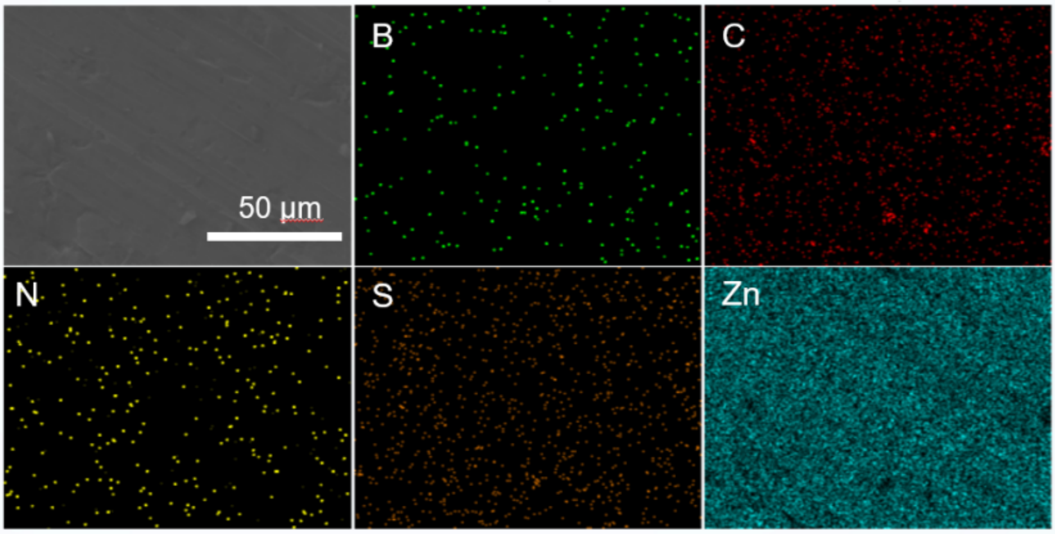

Figure S10. SEM and EDS mapping images of Zn@**CityU-51**.

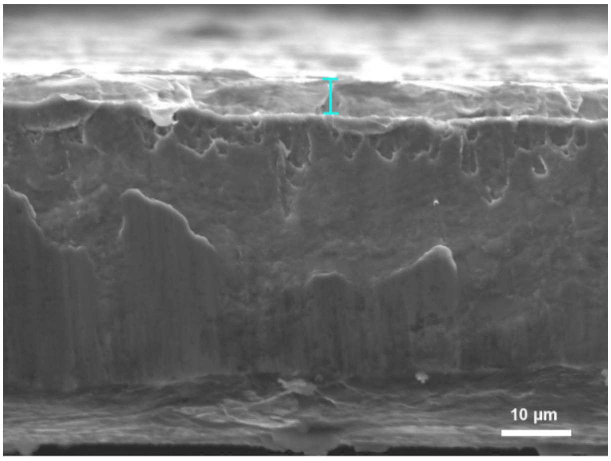

Figure S11. The cross-sectional SEM image of Zn@**CityU-51** anode.

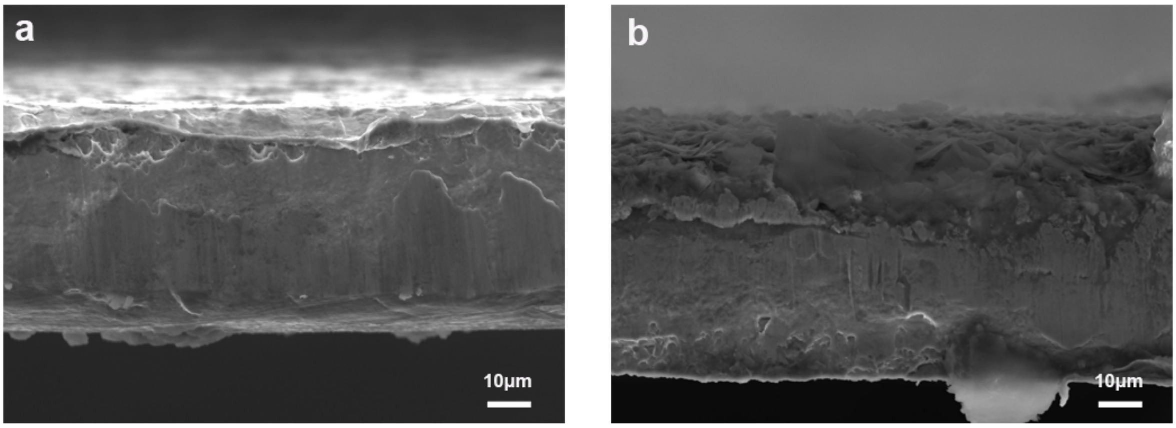

Figure S12. The cross-sectional SEM images of (a) modified Zn metal and (b) blank Zn metal after 8-hour deposition.

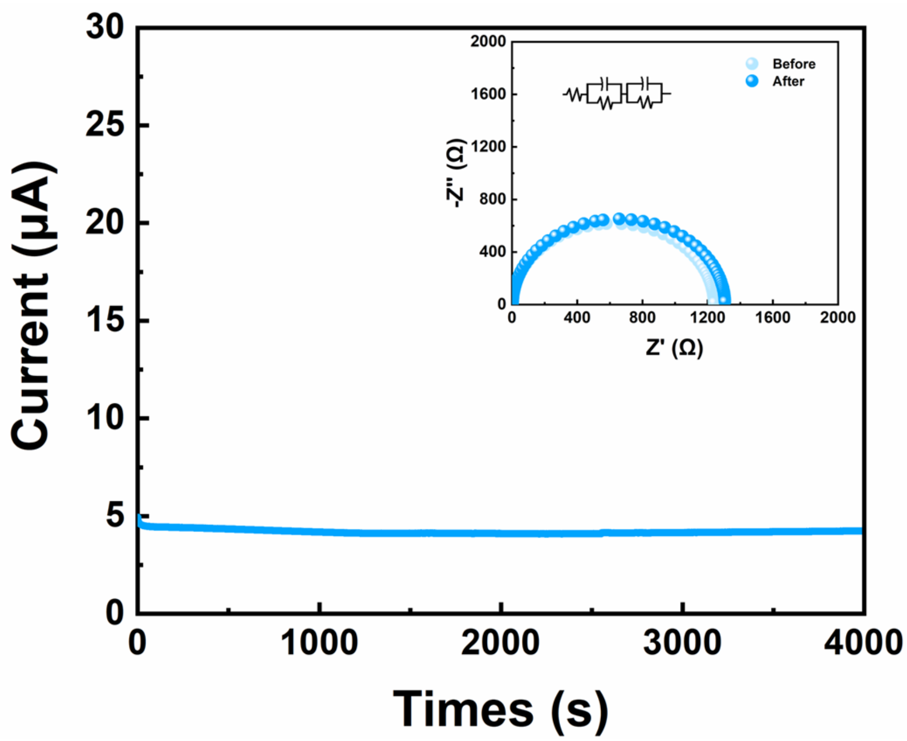

Figure S13. Current-time curves of the Zn@**CityU-51**-Zn symmetric battery with an experimental potential of 20 mV. Inset: The fitting EIS plots of Zn@**CityU-51** electrodes before and after cycling.

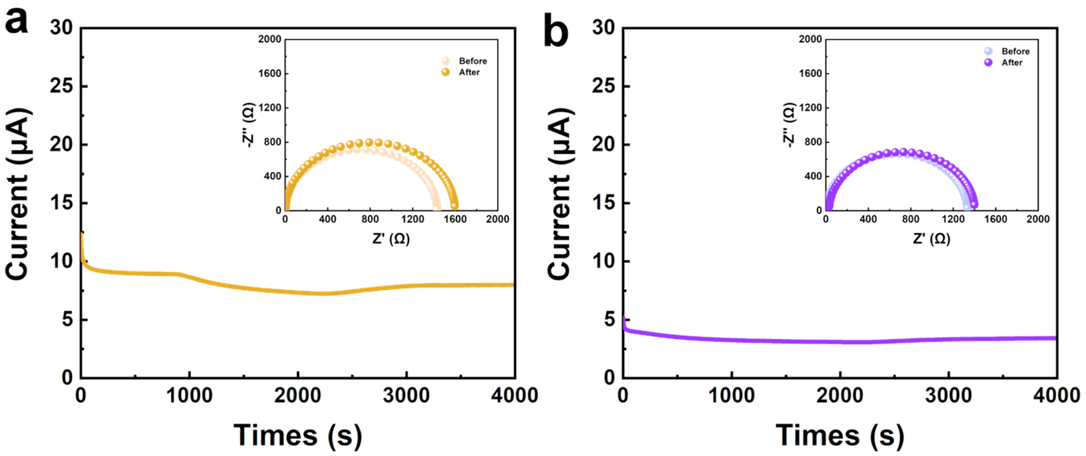

Figure S14. Current-time curves of (a) the Zn-Zn symmetric batteries and (b) the Zn@amorphous polymer-Zn symmetric batteries with an experimental potential of 20 mV. Inset: The fitting EIS plots of the corresponding anodes before and after cycling.

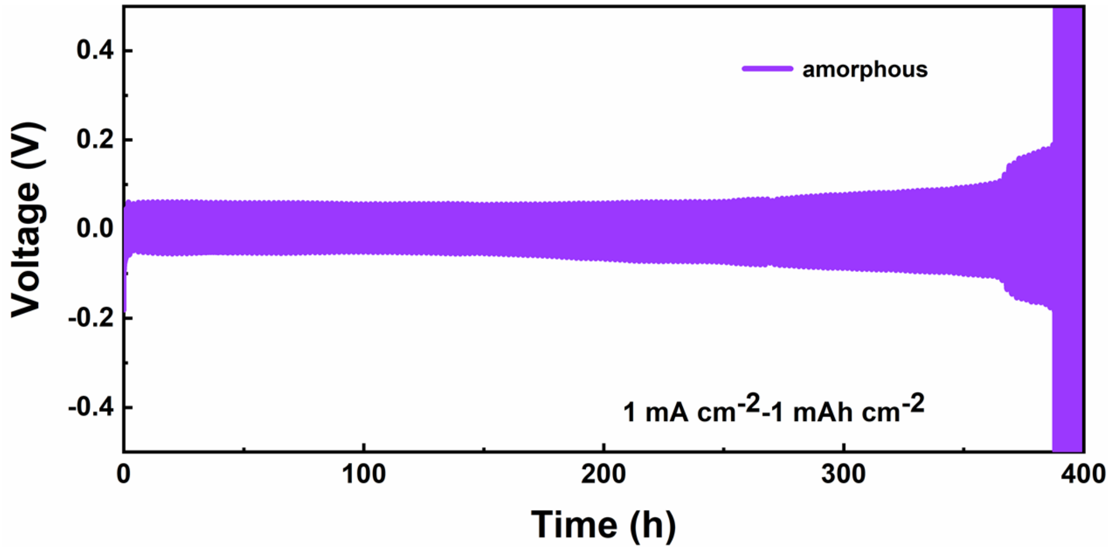

Figure S15. The cycling performance of the batteries using amorphous polymer as the modification layer.

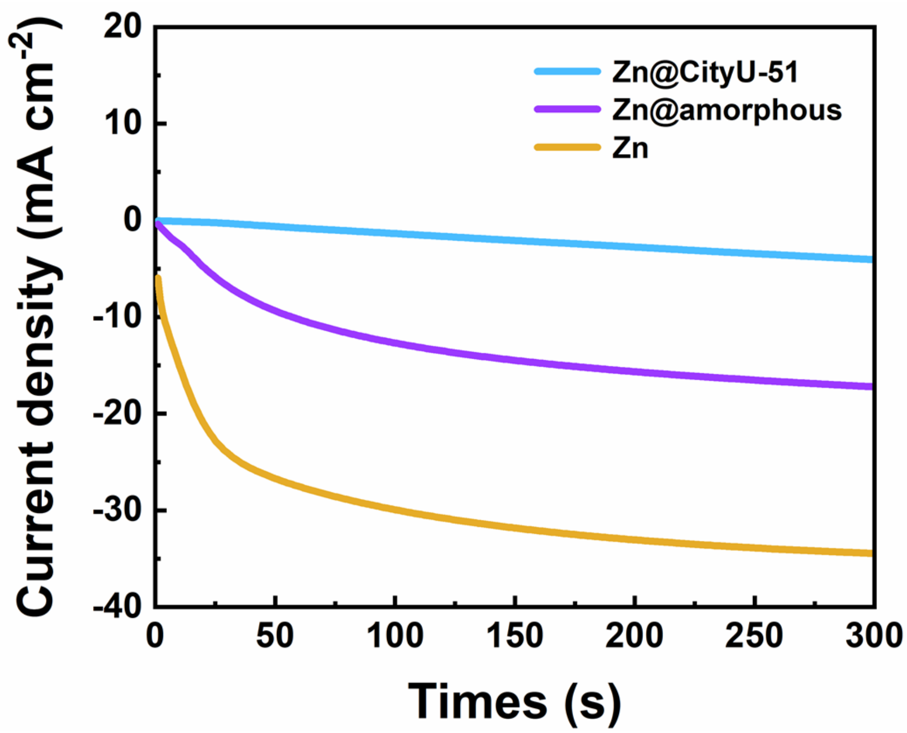

Figure S16. Chronoamperometry profiles of bare Zn, Zn@amorphous and Zn@**CityU-51** symmetric cells at the overpotential of -150 mV.

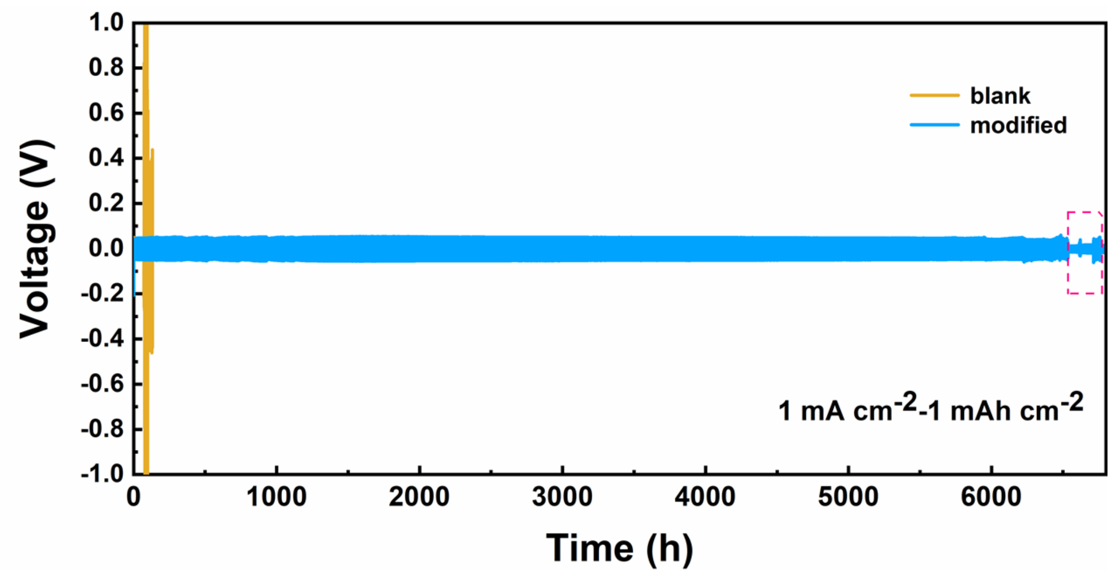

Figure S17. Initial signs of degradation of the Zn@**CityU-51**-Zn batteries operating at 1 mA cm^{-2}.

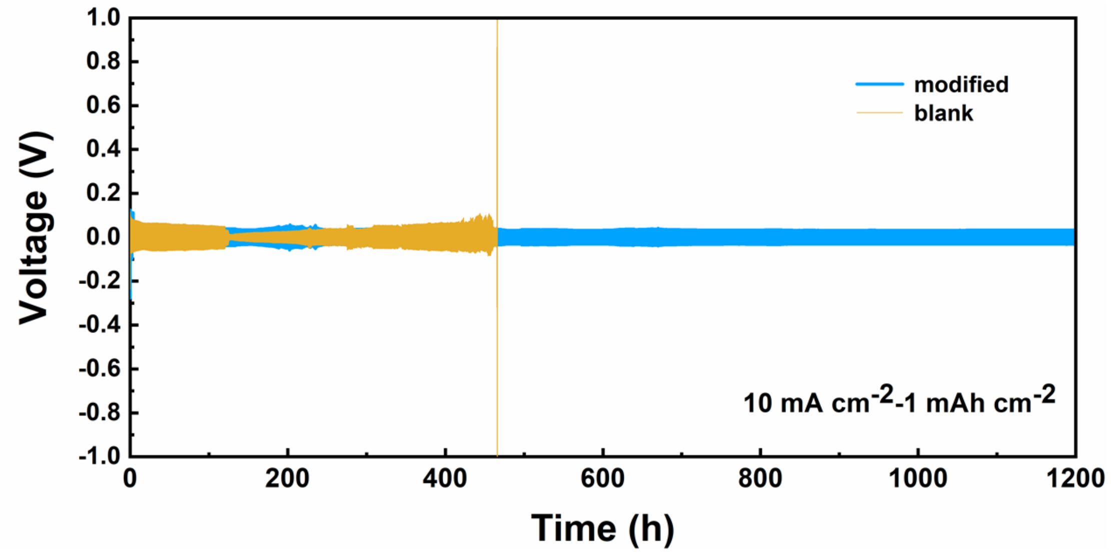

Figure S18. The cycling performance of Zn@**CityU-51**-Zn batteries at 10 mA cm^{-2}.

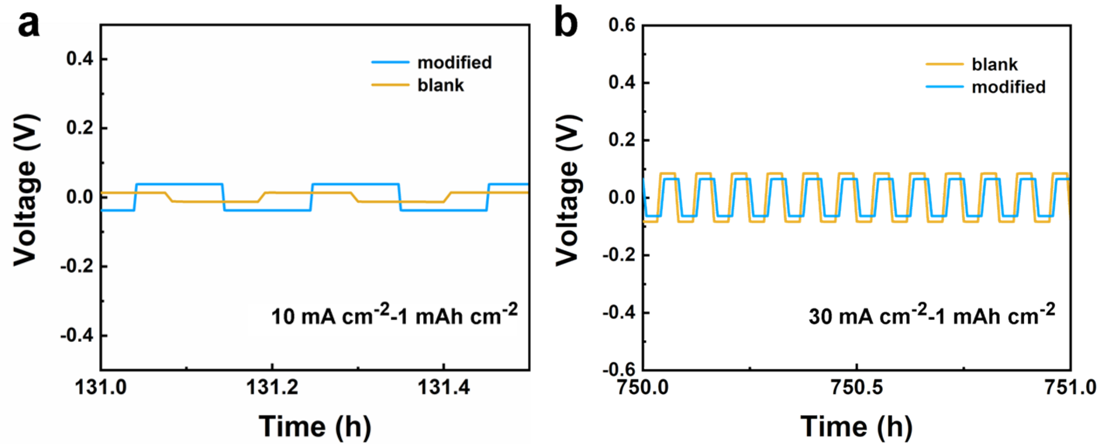

Figure S19. The amplified cycling curves of Zn@**CityU-51**-Zn batteries at (a) 10 mA cm^{-2} and (b) 30 mA cm^{-2}.

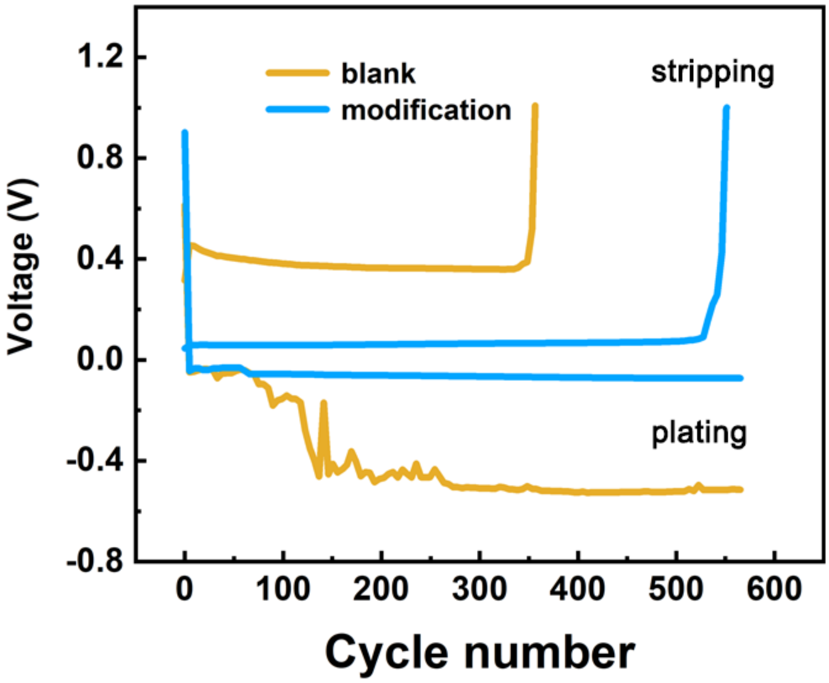

Figure S20. The plating/stripping curves of Zn -Cu and Zn@**CityU-51** -Cu batteries.

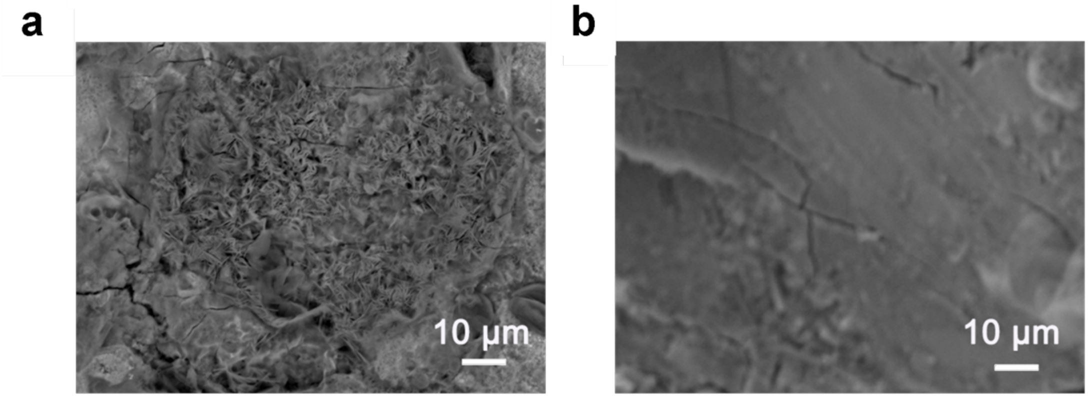

Figure S21. The SEM images of (a) bare and (b) modified Zn metal after cycling in AZIBs.

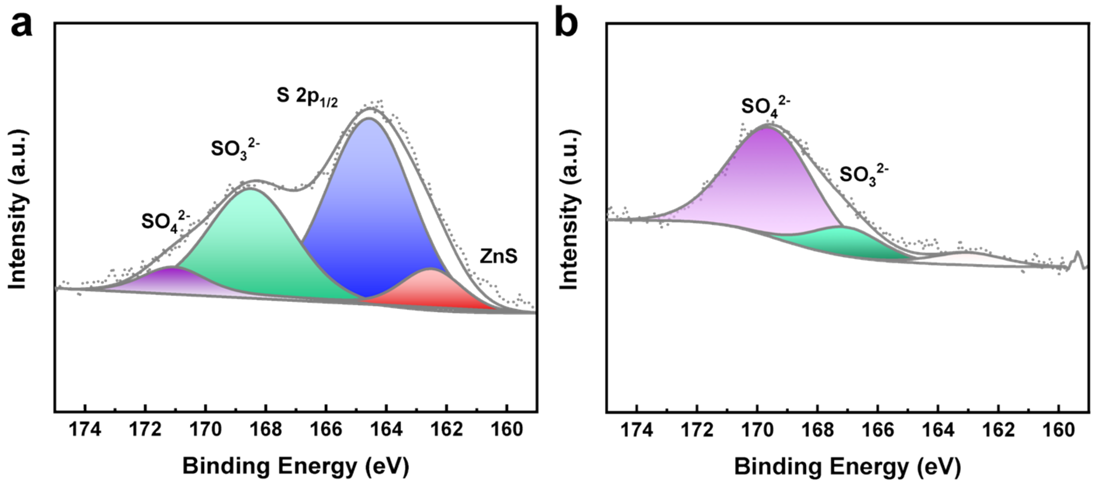

Figure S22. XPS characterizations of (a) Zn@**CityU-51** and (b) bare Zn after cycling.

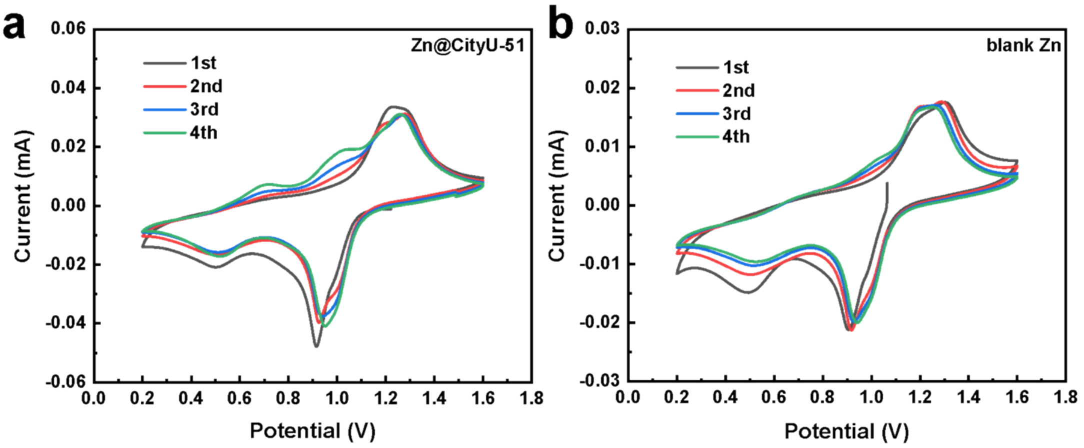

Figure S23. CV curves of (a) V_2O_5//Zn@**CityU-51** and (b) V_2O_5//bare Zn batteries.

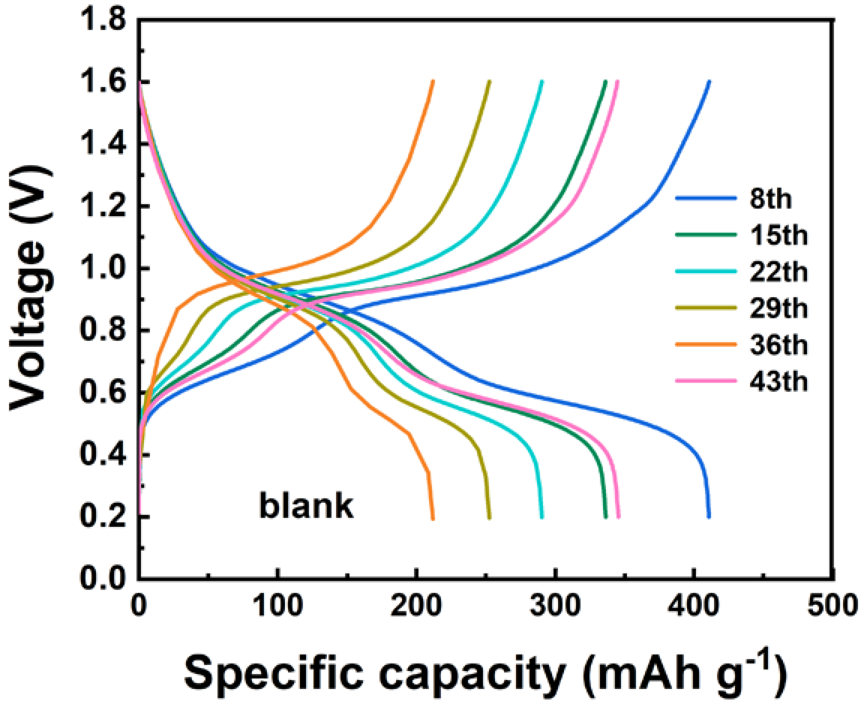

Figure S24. The charging/ discharging profiles of the V_2O_5//bare Zn battery.

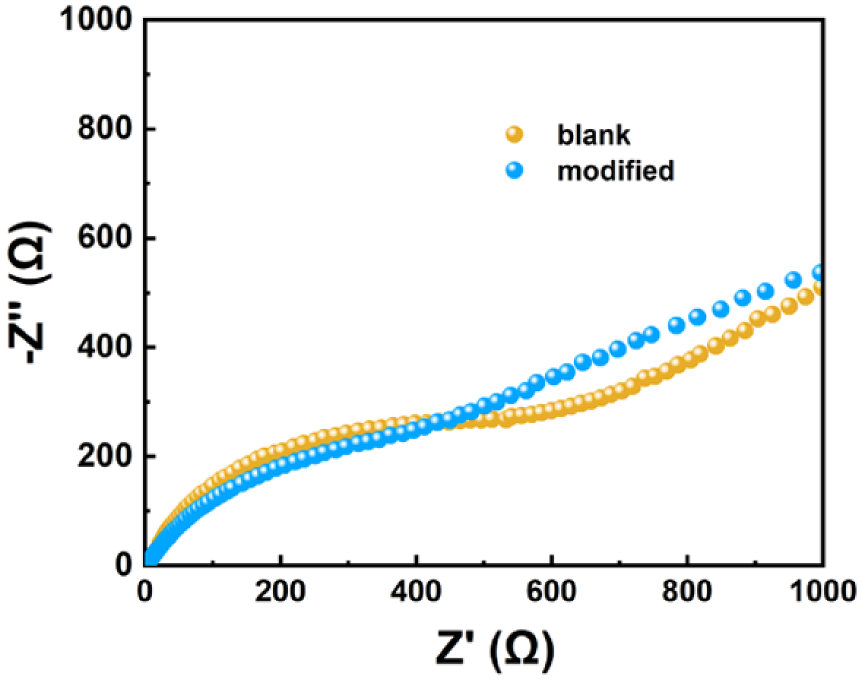

Figure S25. The EIS plots of V_2O_5//Zn@**CityU-51** and V_2O_5//bare Zn batteries after full cycling.

Table S1. Crystal data and structure refinement of **CityU-51** (CCDC 2450236).

	**CityU-51**
**Empirical formula**	$C_{33.5}H_{22}BN_2O_2S_2$
**Formula weight**	559.46
**Crystal system**	monoclinic
**Space group**	P2/c
a (Å)	11.9161(10)
b (Å)	17.3545(10)
c (Å)	14.1611(15)
α (°)	90
β (°)	108.981(11)
γ (°)	90
V (Å^3)	2769.3(4)
Z	4
D_{calc}(g·cm^{-3})	1.342
**Abs.coeff.(mm^{-1})**	2.017
F**(000)**	1060.0
**Reflns collected**	16668
**GOFon** F^2	1.004
R_{int}	0.1274
R_1[a]	0.0901
wR_2**(all data)**[b]	0.2538

[a]$R_1=\Sigma||F_o|- |F_c||/\Sigma|F_o|$. [b]$wR_2= |\Sigma w (|F_o|^2-|F_c|^2)|/\Sigma|w(F_o^2)^2|^{1/2}$

Table S2. Selected bond distance (Å) of **CityU-51**.

Atom1	Atom2	Length/Å
S1	C11	1.762(6)
S1	C10	1.756(5)
S2	C11	1.726(5)
S2	C12	1.742(6)
O1	C26	1.393(7)
O1	B1	1.460(8)
O2	C25	1.371(7)
O2	B1	1.475(7)
N2	C21	1.347(6)
N2	C19	1.314(6)
N2	B1	1.637(7)
C23	B1	1.590(9)
N1	C2	1.294(9)
N1	C1	1.363(10)

[1]2-X,+Y,3/2-Z; [2]1-X,-Y,1-Z

Table S3. Selected bond angles (°) of **CityU-51**.

Atom1	Atom2	Atom3	Angle/°
C10	S1	C11	95.1(3)
C11	S2	C12	96.2(3)
C21	N2	B1	120.0(4)
C19	N2	C21	118.4(4)
C19	N2	B1	120.8(4)
N2	C19	C17	122.8(5)
C13	C12	S2	113.9(4)
C10	C12	S2	117.1(4)
C2	N1	C1	116.6(8)
O1	B1	O2	105.8(5)
O1	B1	N2	106.1(5)
O1	B1	C23	114.3(4)
O2	B1	N2	106.9(4)
O2	B1	C23	115.5(5)

[1]2-X,+Y,3/2-Z; [2]1-X,-Y,1-Z

Table S4. Comparison of the cycling stability of AZIBs between this work and the reported works with various modification strategies.

Modification Strategies	Cycling Stability	Refs.
**CityU-51 layer on Zn**	**1 mA cm^{-2} for 6300 h**<br>**30 mA cm^{-2} for 6000 h**	**This work**
LSPL layer on Zn	1 mA cm^{-2} for 2000 h	[45]
D-mannose and sodium lignosulfonate additives	1 mA cm^{-2} for 6400 h	[46]
CPM additive	2 mA cm^{-2} for 2900 h	[47]
PVDF-SBA15 coating on Zn	3 mA cm^{-2} for 1650 h	[48]
AQS additive	0.5 mA cm^{-2} for 2500 h<br>5 mA cm^{-2} for 1100 h	[49]
ATP layer on Zn	5 mA cm^{-2} for 2800 h	[50]
UMMT layer on Zn	6 mA cm^{-2} for 1300 h	[51]
TPFND additive	10 mA cm^{-2} for 430 h	[52]
ND layer on Zn	1 mA cm^{-2} for 3650 h<br>3 mA cm^{-2} for 2250 h<br>10 mA cm^{-2} for 1016 h	[53]
SCRIS-SALs on Zn	10 mA cm^{-2} for 2500 h	[54]
PMCL on Zn	10 mA cm^{-2} for 5400 h	[55]
Sn layer on Zn	20 mA cm^{-2} for 1200 h	[56]
Pb-PVDF layer on Zn	1 mA cm^{-2} for 8100 h<br>20 mA cm^{-2} for 800 h	[57]
PPPA additive	50 mA cm^{-2} for 400 h	[58]
ABPA additive	50 mA cm^{-2} for 13000 cycles (~260 h)	[59]
